# Genes Associated With Chromatin Modification Within the Swine Placenta Are Differentially Expressed Due to Factors Associated With Season

**DOI:** 10.3389/fgene.2020.01019

**Published:** 2020-09-23

**Authors:** Lea A. Rempel, John J. Parrish, Jeremy R. Miles

**Affiliations:** ^1^USDA, ARS, US Meat Animal Research Center, Clay Center, NE, United States; ^2^Department of Animal Science, University of Wisconsin-Madison, Madison, WI, United States

**Keywords:** DNA methylation, epigenetics, histone modification, placenta, season, swine

## Abstract

Seasonal reproductive inefficiency is still observed in modern swine facilities. We previously reported global placental methylation activity was reduced from summer breedings and tended to be less from semen collected during cooler periods. The objective of the current study was to evaluate chromatin modification marks within swine placenta in relationship to breeding season, semen collection season, and semen storage. White composite gilts were artificially inseminated in August or January using single-sire semen that was collected during warm or cool periods and stored as either cryopreserved or cooled-extended. Gilts were harvested 45 days post-breeding, and placental samples from the smallest, average, and largest fetus in each litter were collected and stored at −80°C until RNA extraction. An RT^2^ Profiler assay featuring 84 known chromatin modification enzyme targets was performed using placental RNA pooled by litter. Real-time quantitative polymerase chain reaction results were analyzed using the MIXED procedure, and *P-*values were Hochberg corrected using the MULTTEST procedure in SAS. The complete model included the fixed effects of breeding season (winter or summer), semen collection season (cool or warm), semen storage (cooled-extended or cryopreserved), interactions; boar as repeated effect; and plate as random effect. If interactions were not significant, only the main effects were tested. The genes, *ATF2*, *AURKA*, and *KDM5B*, were different (*P <* 0.05) by interaction of breeding season, semen collection season, and semen storage. In general, the greatest (*P <* 0.05) expression was in placentas derived from summer breedings. Expression of *AURKA* was also influenced by semen collection and storage. Expression of placental *KDM5B* from winter breedings was also greater (*P <* 0.05) from semen collected during cool periods. Placental expressions of *ASH2L*, *DNMT3B*, *ESCO1*, *HDAC2*, *ING3*, *KDM6B*, *MYSM1*, and *SMYD3* were greater (*P <* 0.05) from summer breedings. Increased expressions of known chromatin modification genes, from placentas derived from summer breedings, are likely responsible for differences in gene transcription between summer- or winter-derived placentas.

## Introduction

Epigenetic modifications, including methylation activity, histone modifications, and miRNA regulation, are essential for appropriate placental development and differentiation throughout pregnancy (Vaiman, 2017). Han et al. (2019) recently reported modification of the H3 histone tail and associated changes in placental gene transcripts in Chinese Meishan swine placenta at midgestation or late gestation. Methylation activity and histone modifications are complex and can be influenced by environmental cues, such as temperature, diet, and pollutants. Feed restriction in gestating gilts resulted in elevated placental percent methylation activity in comparison to control-fed gilts receiving methylating vitamins and docosahexaenoic acid (Lima et al., 2018). Furthermore, those gilts on a restricted diet that received no methylating vitamins or docosahexaenoic acid had reduced litter sizes in contrast to control-fed gilts. Alteration in placental methylation patterns has been associated with impaired fetal growth and early pregnancy loss in humans (Koukoura et al., 2012; Yin et al., 2012). Previously, we have shown differences in placental DNA methylation activity and expression of genes with differentially methylated regions in pregnancies derived from semen collected in cool or warm periods, stored as cooled-extended or cryopreserved, and breeding gilts in summer or winter (Rempel et al., 2019). Specifically, swine placentas derived from winter breedings yielded reduced 5-methylcytosine:5-hydroxymethylcytosine (5mC:5hmC), potentially “loosening” the chromatin structure allowing the transcription of methylation-responsive genes, such as MEST and RHOBTB3 (Rempel et al., 2019).

Placental tissues are derived from the genetic contributions from both the male and female gametes. Modification of gametes and uterine environment due to heat stress may alter the placenta and fetus to the extent of hindering or even precluding pregnancy. Although season has long been known to influence swine fertility and litter size (Wildt et al., 1975; Wettemann and Bazer, 1985; Wettemann et al., 1988), to date, no one has reported the expression of genes that regulate DNA methylation and histone modification activity within the placenta as influenced by male and female contributions. We hypothesized that epigenetic marks (chromatin modification genes) within the placenta will be altered because of semen attributes (collection period or storage) and breeding season (summer or winter). The objective of the current study was to determine if expression of genes, known to regulate histone modifications and DNA methylation pathways, in the placenta was different when generated from semen collected from boars that were exposed to warm or cool periods and stored as cooled-extended or cryopreserved then used for summer or winter breeding of gilts.

## Materials and Methods

All experimental procedures and techniques were reviewed and approved by the US Meat Animal Research Center (USMARC) Animal Care and Use Committee (EO# 5438-31000-091-07). Procedures for handling animals complied with the Guide for the Care and Use of Agricultural Animals in Research and Teaching (FASS, 2010).

### Animal Management and Sample Collection

Semen collection, evaluation, and storage were previously reported (Rempel et al., 2018). Briefly, semen from 12 Duroc boars that met semen quality standards in early June and August 2014 at a commercial boar stud was used in the current study. Semen was stored as either extended and cooled (ExT) or extended then cryopreserved over liquid nitrogen (CRYO) following proprietary methods of the boar stud. All samples were evaluated for motility, morphology, and viability using computer-assisted semen analysis.

Detailed description of gilt care and pregnancy generation were reported (Rempel et al., 2019). Briefly, gilts had previously expressed signs of estrous and were at least 220 days of age at the time of a single-dose insemination following an established altrenogest and chorionic gonadotropin synchronization protocol. Summer breedings occurred in the first week of August 2014, and winter breedings were performed in January 2015. Tissue samples were harvested at 45 days of gestation. A subset of 48 litters generated from 5 boars was selected for the current study, yielding 8 litters from each treatment combination (Table 1).

**TABLE 1:** Basic statistics of the number of litters and treatment group designations.

	**Breeding season**	
**Semen collection**	**Summer**	**Winter**
**Semen storage**		
Warm collection	8	NA
Extended-cooled		
Cool collection	NA	8
Extended-cooled		
Warm collection	8	8
Cryopreserved		
Cool collection	8	8
Cryopreserved		

### RNA Isolation, Pooling, and Transcription of Placental Tissue for Chromatin Modifier RT^2^ Profiler PCR Array

Isolation of RNA from placental tissues was performed on individual placenta samples from the smallest, average, and largest fetus in each litter using Trizol (Thermo Fisher Scientific, Waltham, MA, United States) following the manufacturer’s recommended procedure.

Four hundred fifty nanograms of total placental RNA, pooled by litter, was reverse transcribed into first-strand cDNA using random hexamer and oligo-dT primers for downstream usage with RT^2^ Profiler PCR Arrays following the manufacturer’s recommendations (RT^2^ First Strand Kit, Qiagen, Valencia, CA, United States). A swine-specific Epigenetic Chromatin Modifier Enzyme RT^2^ Profiler PCR Array (RT^2^ array; Qiagen) was used to investigate chromatin modifying gene transcripts within placenta. All RT^2^ array reactions were performed on a Bio-Rad CFX384 real-time polymerase chain reaction (PCR) instrument (Hercules, CA, United States) under the following conditions: 95°C for 10 min followed by 40 cycles at 95°C for 15 s, 60°C for 1 min, with a final melting curve from 65 to 95°C. All samples were void of nontranscribed swine genomic DNA contamination (SGDC; i.e., no detection of SGDC in the RT^2^ array). The positive PCR control (PPC) was consistent across all samples and averaged 22.0 ± 0.22 SD C_T_, within the acceptable parameters for PCR amplification as per manufacturer’s instructions. Average intra-assay CV for PPC was 0.84%, and the interassay CV was 0.99%.

### RNA Isolation, Pooling, and Transcription of Placental Tissue for Validation of Target Genes

Nine hundred nanograms of total placental RNA, pooled by litter, was reverse transcribed into first-strand cDNA using random hexamer and oligo-dT primers following the manufacturer’s recommendations (iScript Reverse Transcription Supermix; Bio-Rad).

Selected gene targets were validated using predesigned swine-specific real-time PrimePCR SYBR Green Assays (qPCR assays; Bio-Rad). Genes selected included chromatin modifiers; ATF2 (qSscCID0012425), ASH2L (qSscCED0011544), and SMYD3 (qSscCID0005185); downstream targets of chromatin modifiers, interleukin 1β (IL-1β) (qSscCED0009600) and NANOG (qSscCED0007337); and a reference gene, GAPDH (qSscCED0017494). Two microliters of cDNA was used in 10 μL real-time PCR reactions according to the manufacturer’s protocol. Samples were ran in triplicate on a Bio-Rad CFX384 real-time PCR instrument under the following conditions: 95°C for 2 min followed by 40 cycles at 95°C for 5 s, 60°C for 30 s, with a final melting curve from 65 to 95°C for 5 s per step.

### Statistical Analyses

All statistical analyses were conducted in SAS 9.4 (SAS Institute Inc., Cary, NC, United States). Data were collected in the linear phase of amplification and then log-transformed into relative quantity (RQ) values. RT^2^ array RQ values and quantitative PCR (qPCR) assay RQ values were analyzed using the MIXED procedure with the fixed effects: collection season (categorically labeled cool or warm), storage of semen (ExT or CRYO), breeding season (summer or winter), and the interaction of collection season by semen storage by breeding season. If interactions were not significant, then only the main effects were tested in the model. In all models, boar was designated as a repeated effect, and dam was random. Stepdown Bonferroni and Hochberg correction for multiple testing were imposed upon the RT^2^ array data using raw *P-*values and MULTTEST procedure in SAS.

The RQ values for the significant transcripts were evaluated for linear relationship with the previously reported global methylation activity (5mC:5hmC) in the placenta (Rempel et al., 2019) using the Pearson correlation analysis in SAS.

## Results and Discussion

External stimuli, both maternal and paternal, have been shown to alter the placental epigenome in multiple species (Deshpande and Balasinor, 2018). We have previously shown altered global methylation activity in the placenta derived from semen collected in cool or warm season and stored as cooled-extended or cryopreserved and then used to inseminate gilts in summer or winter (Rempel et al., 2019). Briefly, placenta derived from winter breedings had reduced 5mC:5hmC activity. Additionally, differences were detected in placental expression of genes with differentially methylated regions or genes responsive to methylation status due to semen collection, semen storage traits, and breeding season (Rempel et al., 2019). In the current study, we used an epigenetic chromatin modifier RT^2^ profiler array to determine regulatory epigenetic components in swine placenta that were altered by breeding season or semen characteristics. Of the 84 target genes tested, 81 genes produced a detectable product (Supplementary Table 1). Thirty-two genes were nominally different (*P <* 0.05) prior to stepdown Bonferroni or Hochberg correction testing.

**FIGURE 1:**
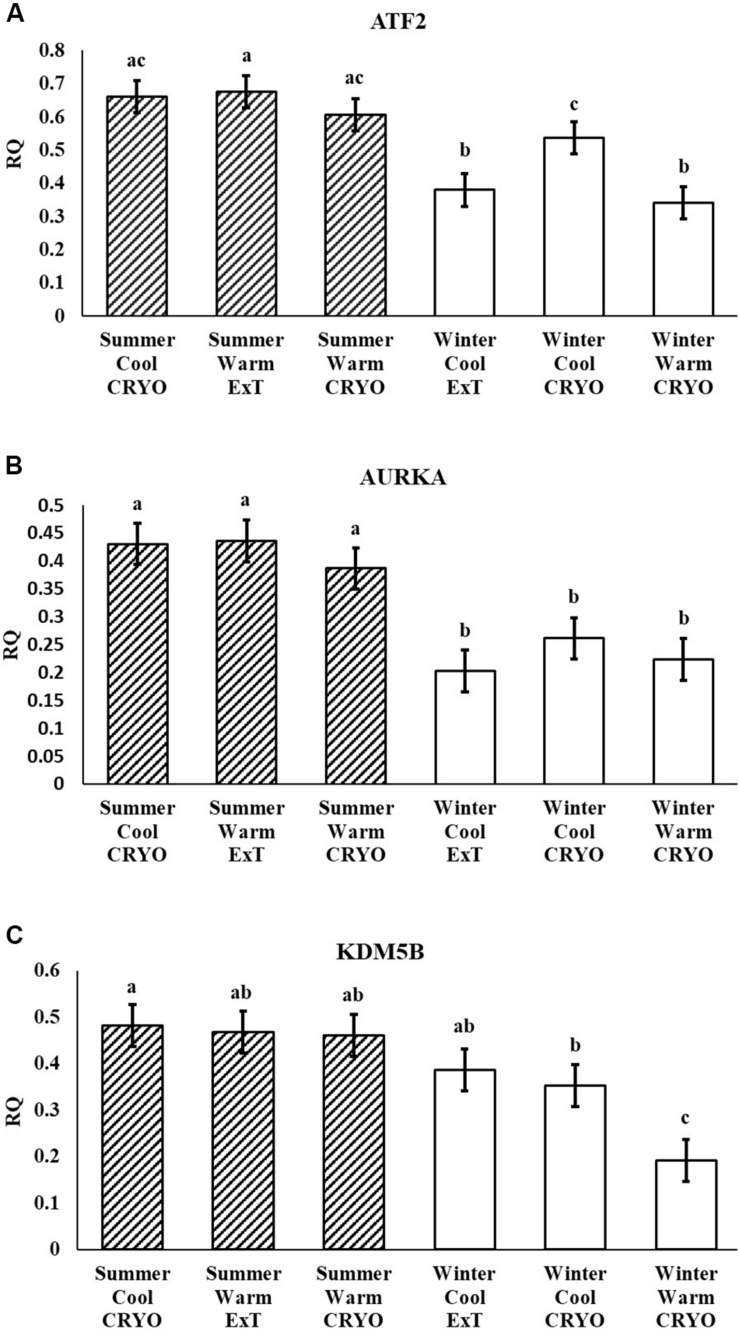
Relative expression (RQ) of placental ATF2 **(A)**, AURKA **(B)**, and KDM5B **(C)** by RT^2^ Profiler Array. Interaction effect of breeding season (summer or winter), semen collection season (cool or warm), and semen storage [cryopreserved (CRYO) or cooled-extended (ExT)]. Means reported as least-squares means ± SE, and bars with different letters differ by *P <* 0.05.

**FIGURE 2:**
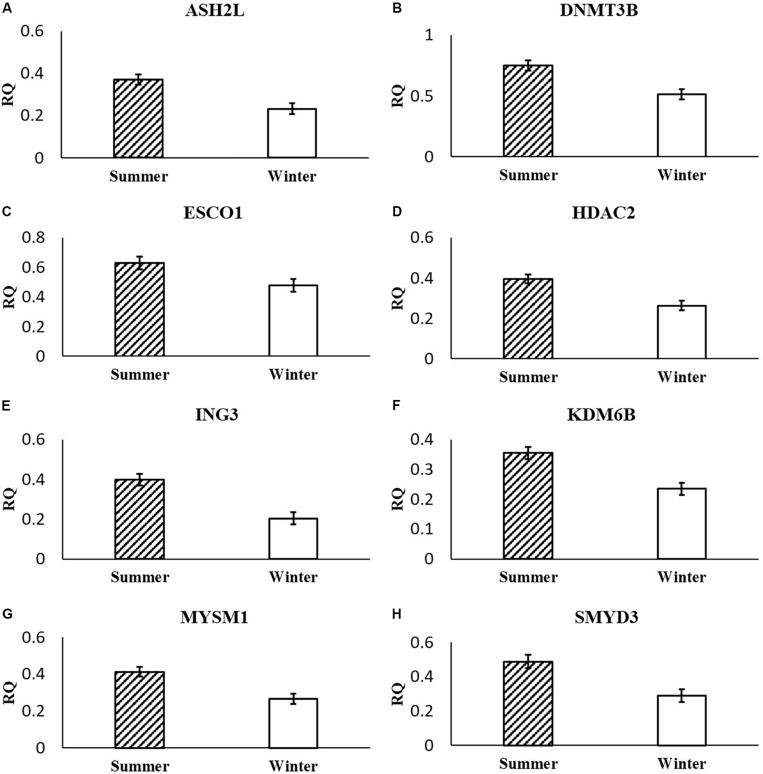
Relative expression (RQ) of placental ASH2L **(A)**, DNMT3B **(B)**, ESCO1 **(C)**, HDAC2 **(D)**, ING3 **(E)**, KDM6B **(F)**, MYSM1 **(G)**, and SMYD3 **(H)** that differ by breeding season (Summer or Winter) using the RT^2^ Profiler Array. Means reported as least square means ± SE and differ by *P* < 0.05.

Following multiple testing corrections, three genes were significant (*P <* 0.05; Figure 1) for the interaction of breeding season, semen collection season, and semen collection storage. Expression was different for histone acetyltransferase, activating transcription factor 2 (ATF2); histone phosphorylate, Aurora kinase A (AURKA); and DNA and histone demethylase, lysine demethylase 5B (KDM5B). In addition to the three gene products with interaction effects, eight genes were significant following correction for multiple testing (*P <* 0.05; Figure 2) for the main effect of breeding season. Specifically, relative expression of these genes was greater (*P <* 0.05) in the placenta derived from summer breedings in contrast to winter breedings (Figure 2). The transcripts were stratified among several DNA methylation and histone modification categories including a DNA methyltransferase (DNMT), DNMT3B; a histone acetyltransferase, establishment of sister chromatid cohesion N-acetyltransferase 1 (ESCO1); histone methyltransferases, SET and MYND domain containing 3 (SMYD3), ASH2-like, histone methyltransferase complex subunit (ASH2L), and inhibitor of growth family member (ING) 3; a histone deubiquitination protein, Myb Like, SWIRM, and MPN Domains 1 (MYSM1); a DNA and histone demethylase, lysine demethylase 6B (KDM6B); and a histone deacetylase (HDAC), HDAC2. Pearson correlations were performed to determine if a linear relationship existed between chromatin modification genes and placental 5mC:5hmC activity. Only relative expression of AURKA had a significant association (*P <* 0.01).

**FIGURE 3:**
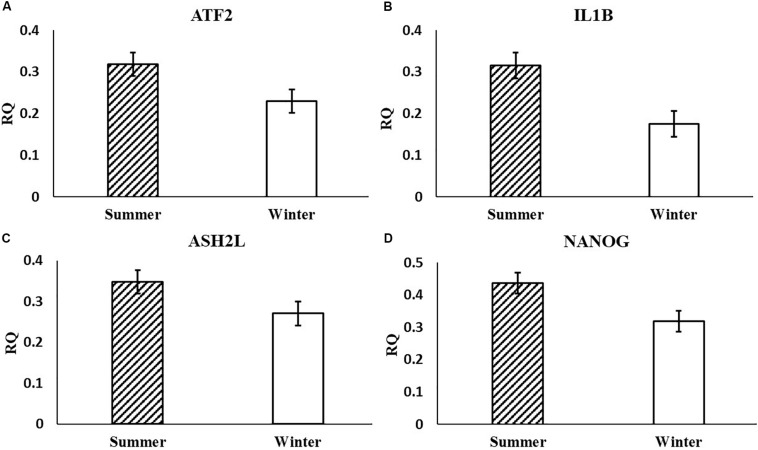
Relative expression (RQ) of placental ATF2 **(A)**, IL1B **(B)**, ASH2L **(C)**, and NANOG **(D)** that differ by breeding season (summer or winter) using qPCR assays. Means reported as least-squares means ± SE and differ by *P* < 0.05.

**FIGURE 4:**
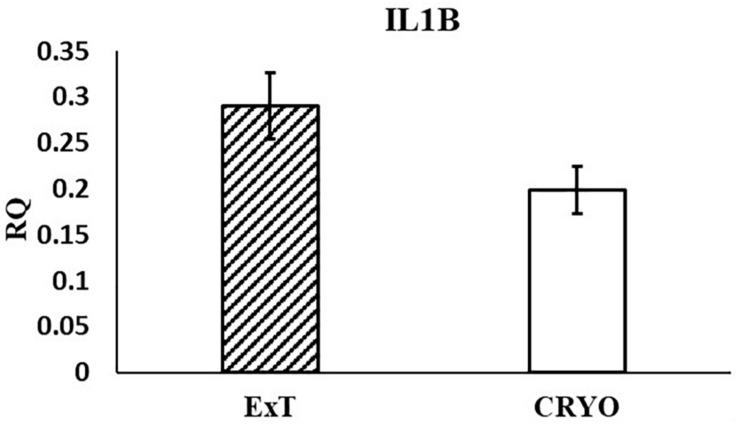
Relative expression (RQ) of placental ILIB by semen storage (Cooled-Extended; ExT or Cryopreserved; CRYO) using qPCR Assays. Means reported as least square means ± SE and differ by *P* < 0.05.

In the current study, the leucine zipper family of DNA-binding proteins, ATF2, had greater (*P* = 0.0160) relative expression in the placenta from summer breedings and least in the placenta from winter breedings that were generated from semen from cool collection and cooled-extended or warm collection and then cryopreserved (Figure 1A). Not only is ATF2 a DNA-binding protein, it also can become a histone acetyltransferase upon phosphorylation, acetylating histone tails, H2B and H4 (Kawasaki et al., 2000). Acetylation generally relaxes chromatin structure, increasing gene transcription. These data suggest that placenta derived from summer breedings has the potential for increased chromatin relaxation eliciting increased gene transcription, while ATF2 gene expression in the placenta generated from winter breedings may be more influenced by paternal contributions, specifically semen collection season and storage methods. Validation of relative expression of placental ATF2 using qPCR assays indicated a breeding season effect only (Figure 3A). Placentas derived from summer breedings yielded greater (*P* = 0.0311) relative expression of ATF2 in comparison to winter breedings. Interestingly, immunoprecipitation studies indicated that ATF2 binds with several HDACs including HDAC2, likely working to regulate activity of ATF2 as a histone acetylate (Liu et al., 2013). Within the current study, HDAC2 had greater (*P* = 0.0234) relative expression in the placenta from summer breedings in comparison to winter breedings (Figure 2D). Whether the increase in placental relative expression of HDAC2 is directly or indirectly a result of relative expression of ATF2 and if these two proteins are interacting with each other to modify placental chromatin activity is still to be determined. Similarly, IL-1B is also a downstream target of ATF2 (Reimold et al., 2001). In the current study, qPCR assays verified that relative expression of IL-1B in the placenta was also greater (*P* = 0.0027) from summer breedings (Figure 3B). But IL-1B relative expression was also greater (*P* = 0.0437) in the placenta as a result of using ExT semen for breeding in comparison to CRYO semen (Figure 4). In addition to serving as an epigenetic regulator, ATF2 may also be acting upon other downstream targets in response to stress.

The relative expression of AURKA in swine placenta was different (*P* = 0.0081) by an interaction among breeding season, season of semen collection, and semen storage, but the overall consensus was greater relative expression from summer breedings than winter breedings (Figure 1B). From an epigenetic perspective, AURKA phosphorylates serine and threonine residues on the H3 histone tail; however, limited information is available on the exact epigenetic role of AURKA. Liu et al. (2016) verified AURKA as a candidate gene in gastric cancer and presented data to support the indirect role of AURKA as an epigenetic modifier in gastric cancer. Upregulation of AURKA in gastric cancer led to increased activation of Wnt and Akt signaling pathways that subsequently induced histone modifications to the promoter region of the Twist gene (Liu et al., 2016). Activation of Twist leads to increased epithelial–mesenchymal transition. The dynamic transition of cells during placentation is often compared to the cellular changes during cancer. Trophoblast cells are rapidly modified during placentation and transitioning from epithelial-to-mesenchymal cells. In our study, the increased relative expression of AURKA from summer breedings may either be functioning (1) as a chromatin modifier (i.e., histone phosphorylation), (2) to activate signaling pathways (e.g., Wnt and Akt) through phosphorylation that subsequently induce secondary histone modifications, or (3) a combination (histone phosphorylation and phosphorylation and activation of target pathways) to support placental development during environmental stress, summer breedings.

To evaluate the relationship between the previously reported placental global methylation activity (Rempel et al., 2019), Pearson correlation analysis was performed. A positive correlation (*r*^2^ = 0.38, *P* = 0.0093) between the relative expression of AURKA and placental global methylation activity (5mC:5hmC) was observed. In the previous publication, placental tissues from summer breedings had increased 5mC:5hmC activity, suggesting a reduction in methylation-responsive gene transcription activation. Our current findings support these data in which increased levels of AURKA may be acting to modify placental structure and function through phosphorylation pathways instead of methylation-responsive targets.

Within our study, placental expression of KDM5B was least (*P* = 0.0474) in winter breedings using cryopreserved semen collected during a warm month, whereas winter breedings using cooled-extended semen or cryopreserved semen collected during a cool month were generally intermediary to breedings that occurred in summer (Figure 1C). Interestingly, KDM5B regulates H3K4 methylation within trophoblast stem cells influencing trophoblast lineage into placental tissue. Specifically, reduction of KDM5B led to downregulation of self-renewal of trophoblast stem cells and increased differentiation of those cells into placental lineage (Xu and Kidder, 2018). Another lysine demethylase, KDM6B, had greater (*P* = 0.0080) relative expression in the placenta from summer breedings in contrast to winter breedings (Figure 2F). Lysine demethylase 6B demethylates H3K27 sites, thereby activating HOX gene expression (Agger et al., 2007; Lan et al., 2007). HOX genes and regulated methylation are necessary for proper placental development (Scotti and Kmita, 2012; Novakovic et al., 2017). Greater expression of KDM5B and KDM6B within the placenta from summer breedings may be acting to augment or enhance placental development, specifically trophoblast activity.

In the current study, ASH2L, a histone methyltransferase at the H3K4 residue, had greater (*P* = 0.0380) relative placental expression from summer breedings in comparison to winter breedings using the RT^2^ Profiler Array (Figure 2A) and tended (*P* = 0.0648) to have greater relative expression from the validation qPCR assay (Figure 3C). Typically, chromatin methylation is associated with reduced transcription activity. However, methylation of H3K4 has been proposed as counter to H3K9- and H3K27-methylation chromatin repressive activity (Shilatifard, 2006). Furthermore, ING3, a histone acetyltransferase, interacts with methylated H3K4 residues as a member of a larger complex of interacting proteins, initiating acetylation and thereby relaxing the chromatin structure (Doyon et al., 2006). Data from the RT^2^ Profiler Array indicated placentas derived from summer breedings had greater (*P* = 0.0073) relative expression of ING3 in comparison to winter breedings (Figure 2E). Pontin and reptin, two proteins within the larger protein complex that contains ING3, are known to regulate hypoxia signaling (Lee et al., 2011). Placental expression of ASH2L and ING3 from summer breedings may be acting in concert with other proteins to alter chromatin structure enhancing downstream transcription activity to offset seasonal adversity.

DNMT methylates cytosine residues, which generally reduces transcription activity. In the current study, DNMT3B relative expression was greater (*P* = 0.0308) from summer breedings in comparison to winter breedings (Figure 2B). Grazul-Bilska et al. (2011) evaluated expression of DNMTs within sheep placenta from day 16 to day 30 of pregnancy. Expression of DNMT3B in sheep placenta decreased as pregnancy progressed. *In vitro* studies using human embryonic stem cells differentiating into trophoblast cells indicated expression of DNMT3B was down-regulated and verified in trophoblast cells from human placenta samples (Sarkar et al., 2016).

The histone acetyltransferase, ESCO1, had decreased (*P* = 0.0158) relative expression in the placenta from winter breedings in comparison to placenta from summer breedings (Figure 2C). In bladder cancer tissue, ESCO1 was overexpressed in contrast to adjacent normal tissues, and knockdown studies of ESCO1 reduced growth, migration, and invasion of bladder cancer cells (Zhang et al., 2016). Placenta cells have similarities to cancer cells. The differences in ESCO1 molecular profile between summer- or winter-derived placentas may be an artifact of cellular adjustments to support placental growth development.

The histone deubiquitinase, MYSM1, had greater (*P* = 0.0450) placental relative expression in the placenta from summer breedings in contrast to winter breedings (Figure 2G). MYSM1 coordinates histone acetylation and deubiquitination at H2A domains and is necessary for activation of promoters such as androgen receptor (Zhu et al., 2007). Placental androgen receptor in ovine co-immunoprecipitated with lysine demethylases, KDM1A and KDM4D, and was thought to regulate placental vascular endothelial growth factor A (VEGFA) expression during placental development (Cleys et al., 2015). The placenta is highly influenced by members of the VEGF family, and epigenetic modifications may be acting to alter the placental vascular infrastructure to support or sustain fetal life during summer conditions.

Histone methyltransferases methylate targets on histone tails. SET and MYND 3 can methylate H3K4, H4K5, and H4K20 residues (Hamamoto et al., 2004; Foreman et al., 2011; Van Aller et al., 2012). Within the current study, summer-derived placentas had greater (*P* = 0.0450) relative expression of SMYD3 in comparison to placentas from winter breedings (Figure 2H). SET and MYND 3 can also methylate nonhistone proteins, such as VEGFR1 (Kunizaki et al., 2007). Previous reports have shown that methylation of VEGFR1 was negatively correlated to expression of VEGFR1 (Kim et al., 2009). As previously mentioned, the VEGF family is ubiquitously expressed in the placenta and has angiogenic and vasculogenic function. The differences between placenta derived from summer breedings and those from winter breedings may require subtle changes to these systems for overall health and support. Although we did not evaluate any heat shock proteins (HSPs), it is known that HSP90 can enhance SMYD3 activity (Hamamoto et al., 2004). Placentas generated in summer months may be receiving molecular signals to augment development. Validation of SMYD3 using independent qPCR assays did not confirm increased placental relative expression in summer versus winter. A downstream SMYD3-responsive gene, NANOG, however, was verified by qPCR assays to have greater (*P* = 0.0145) relative expression in the placenta from summer breedings in contrast to winter breedings (Figure 3D). Suppression of SMYD3 in embryonic cell lineage has been shown to constitutively downregulate expression of NANOG and decrease embryo attachment and development (Suzuki et al., 2015; Bai et al., 2016). In the current study, increased relative expression of SMYD3 by RT^2^ Profiler Array and validation of the downstream target, NANOG, by qPCR assays in placentas from summer breedings versus winter breedings suggest extraembryonic lineage cells are actively modifying expression to adjust to seasonal differences.

Even under the best circumstances, epigenetic activity is required to develop healthy placenta (Rahnama et al., 2006). In the current study, we demonstrate that season of breeding was the primary factor altering relative expression of chromatin modification components making the placenta a pliable tissue that provided an increased opportunity for fetal survival. Actions of the chromatin modification genes likely coordinated gene transcription or repression to fine tune placental development and activity. Interestingly, the increased placental chromatin modification transcripts from summer breedings may also be functioning to maintain increased levels of global 5mC:5hmC in the placenta as previously determined (Rempel et al., 2019), relying more on alternate epigenetic mechanisms, such as histone modifications. Further investigations into the specific chromatin modification components along with interacting proteins and downstream targets will provide a more defined network of action within the placenta, leading to reduction in pregnancy losses during the warm breeding season.

## Data Availability Statement

The raw data supporting the conclusions of this article will be made available by the authors, without undue reservation.

## Ethics Statement

The animal study was reviewed and approved by USDA/ARS/US Meat Animal Research Center Institutional Animal Care and Use Committee.

## Author Contributions

LR designed and carried out the experiments and wrote the manuscript. JP assessed the semen and provided student and editorial support. JM assisted with the design and experiments and performed statistical analyses. All authors contributed to the article and approved the submitted version.

## Disclaimer

Mention of trade names is necessary to report factually on available data; however, the USDA neither guarantees nor warrants the standard of the product, and the same by USDA implies no approval of the product to the exclusion of others that may also be suitable.

## Conflict of Interest

The authors declare that the research was conducted in the absence of any commercial or financial relationships that could be construed as a potential conflict of interest.
