# Supplementary material for: Genes Associated With Chromatin Modification Within the Swine Placenta Are Differentially Expressed Due to Factors Associated With Season
Source: Front Genet. 2020 Sep 23;11:1019. doi: 10.3389/fgene.2020.01019 (PMC7538786; doi:10.3389/fgene.2020.01019)
Supplement: Supplementary file 1 [file Table_1.docx]

**Supplementary Table 1. List of gene targets on the RT^2^ Chromatin Modification Enzymes Profiler Array (Qiagen).**

| GenBank | Symbol | Description | Location |
| --- | --- | --- | --- |
| XM_003125708 | ASH1L | Probable histone-lysine N-methyltransferase ASH1L-like | A1 |
| NM_001243568 | ASH2L | Ash2 (absent, small, or homeotic)-like (Drosophila) | A2 |
| XM_003133491 | ATF2 | Activating transcription factor 2 | A3 |
| NM_001025225 | AURKA | Aurora kinase A | A4 |
| NM_213919 | AURKB | Aurora kinase B | A5 |
| XM_003354497 | BAZ1B | Bromodomain adjacent to zinc finger domain, 1B | A6 |
| XM_003123208 | CARM1 | Coactivator-associated arginine methyltransferase 1 | A7 |
| XM_001927509 | CDYL | Chromodomain protein, Y-like | A8 |
| XM_005662139 | CIITA | Class II, major histocompatibility complex, transactivator | A9 |
| XM_001924382 | CSRP2BP | CSRP2 binding protein | A10 |
| XM_003121437 | CXXC1 | CXXC finger protein 1 | A11 |
| NM_001032355 | DNMT1 | DNA (cytosine-5-)-methyltransferase 1 | A12 |
| NM_001097437 | DNMT3A | DNA (cytosine-5-)-methyltransferase 3 alpha | B1 |
| XM_001928593 | DNMT3B | DNA (cytosine-5-)-methyltransferase 3 beta | B2 |
| XM_005674632 | DOT1L | DOT1-like histone H3K79 methyltransferase | B3 |
| XM_005670237 | DZIP3 | DAZ interacting protein 3, zinc finger | B4 |
| NM_001101823 | EHMT2 | Euchromatic histone-lysine N-methyltransferase 2 | B5 |
| XM_001929213 | EP300 | E1A binding protein p300 | B6 |
| XM_003130718 | EPC1 | Enhancer of polycomb homolog 1-like | B7 |
| XM_003127852 | ESCO1 | Establishment of cohesion 1 homolog 1 (S. cerevisiae) | B8 |
| A XM_003483396 | ESCO2 | Establishment of sister chromatid cohesion N-acetyltransferase 2 | B9 |
| NM_001244309 | EZH2 | Enhancer of zeste homolog 2 (Drosophila) | B10 |
| XM_003354838 | FBXO11 | F-box protein 11 | B11 |
| XM_005671969 | HAT1 | Histone acetyltransferase 1 | B12 |
| XM_003126776 | HDAC10 | Histone deacetylase 10-like | C1 |
| XM_005669815 | HDAC11 | Histone deacetylase 11 | C2 |
| XM_001925318 | HDAC2 | Histone deacetylase 2 | C3 |
| NM_001243827 | HDAC3 | Histone deacetylase 3 | C4 |
| XM_005657593 | HDAC4 | Histone deacetylase 4 | C5 |
| XM_003360315 | HDAC6 | Histone deacetylase 6 | C6 |
| XM_005667668 | HDAC9 | Histone deacetylase 9 | C7 |
| NM_001244268 | ING3 | Inhibitor of growth family, member 3 | C8 |
| XM_003131405 | KAT2A | K(lysine) acetyltransferase 2A | C9 |
| XM_003358330 | KAT2B | K(lysine) acetyltransferase 2B | C10 |
| NM_001243915 | KAT5 | K(lysine) acetyltransferase 5 | C11 |
| XM_005674537 | KAT6A | K(lysine) acetyltransferase 6A | C12 |
| XM_001928949 | KAT6B | K(lysine) acetyltransferase 6B | D1 |
| XM_003124470 | KAT8 | K(lysine) acetyltransferase 8 | D2 |
| NM_001112687 | KDM1A | Lysine (K)-specific demethylase 1A | D3 |
| XM_005668019 | KDM5B | Lysine (K)-specific demethylase 5B | D4 |
| NM_001097433 | KDM5C | Lysine (K)-specific demethylase 5C | D5 |
| XM_005657029 | KDM6B | Lysine (K)-specific demethylase 6B | D6 |
| XM_003357320 | KMT2A | Myeloid/lymphoid or mixed-lineage leukemia (trithorax homolog, Drosophila) | D7 |
| XM_005654261 | KMT2C | Lysine (K)-specific methyltransferase 2C | D8 |
| XM_005667720 | KMT2E | Lysine (K)-specific methyltransferase 2E | D9 |
| XM_003126953 | LOC100512 284 | SET domain containing 6 | D10 |
| XM_003360365 | LOC100523 762 | Histone deacetylase 8-like | D11 |
| XM_003355640 | LOC100627 559 | Histone deacetylase 7-like | D12 |
| XM_005658916 | LOC767626 | Suppressor of variegation 3-9-like protein 1 | E1 |
| XM_005674561 | MBD2 | Methyl-CpG binding domain protein 2 | E2 |
| XM_003126111 | MLL2 | Myeloid/lymphoid or mixed-lineage leukemia 2 | E3 |
| XM_003127968 | MYSM1 | Myb-like, SWIRM and MPN domains 1 | E4 |
| NM_001195359 | MYST2 | K(lysine) acetyltransferase 7 | E5 |
| NM_001025228 | NCOA1 | Nuclear receptor coactivator 1 | E6 |
| NM_001114276 | NCOA3 | Nuclear receptor coactivator 3 | E7 |
| XM_003483942 | NCOA6 | Nuclear receptor coactivator 6 | E8 |
| XM_003122163 | NEK6 | NIMA (never in mitosis gene a)-related kinase 6 | E9 |
| XM_003123667 | NSD1 | Nuclear receptor binding SET domain protein 1 | E10 |
| XM_005667164 | PAK1 | P21 protein (Cdc42/Rac)-activated kinase 1 | E11 |
| XM_003355999 | PRMT1 | Protein arginine methyltransferase 1 | E12 |
| XM_005658906 | PRMT2 | Protein arginine methyltransferase 2 | F1 |
| NM_001160093 | PRMT5 | Protein arginine methyltransferase 5 | F2 |
| NM_001190183 | PRMT6 | Protein arginine methyltransferase 6 | F3 |
| XM_003126909 | PRMT7 | Protein arginine N-methyltransferase 7-like | F4 |
| XM_003481700 | PRMT8 | Protein arginine methyltransferase 8 | F5 |
| XM_003354538 | RNF40 | E3 ubiquitin-protein ligase BRE1B-like | F6 |
| XM_003484085 | RPS6KA3 | Ribosomal protein S6 kinase, 90kDa, polypeptide 3 | F7 |
| XM_005655086 | SETD1A | SET domain containing 1A | F8 |
| XM_005670628 | SETD1B | SET domain containing 1B | F9 |
| XM_005669478 | SETD2 | SET domain containing 2 | F10 |
| XM_001925288 | SETD3 | SET domain containing 3 | F11 |
| XM_003358943 | SETD4 | SET domain containing 4 | F12 |
| XM_001927800 | SETD5 | SET domain containing 5 | G1 |
| XM_005656533 | SETD7 | SET domain containing (lysine methyltransferase) 7 | G2 |
| XM_005670605 | SETD8 | SET domain containing (lysine methyltransferase) 8 | G3 |
| XM_005663486 | SETDB1 | SET domain, bifurcated 1 | G4 |
| XM_003130966 | SETDB2 | Calcium binding protein 39-like | G5 |
| NM_001160089 | SMYD1 | SET and MYND domain containing 1 | G6 |
| NM_001160092 | SMYD3 | SET and MYND domain containing 3 | G7 |
| XM_003122431 | SUV420H1 | Histone-lysine N-methyltransferase SUV420H1-like | G8 |
| XM_001927284 | UBE2A | Ubiquitin-conjugating enzyme E2A | G9 |
| NM_001257356 | UBE2B | Ubiquitin-conjugating enzyme E2B | G10 |
| XM_003358897 | USP16 | Ubiquitin carboxyl-terminal hydrolase 16-like | G11 |
| XM_003128809 | WHSC1 | Probable histone-lysine N-methyltransferase NSD2-like | G12 |
| XM_003357928 | ACTB | Actin, beta | H1 |
| NM_213978 | B2M | Beta-2-microglobulin | H2 |
| NM_001206359 | GAPDH | Glyceraldehyde-3-phosphate dehydrogenase | H3 |
| NM_001032376 | HPRT1 | Hypoxanthine phosphoribosyltransferase 1 | H4 |
| NM_001244068 | RPL13A | Ribosomal protein L13a | H5 |
| SA_00133 | SGDC | Pig Genomic DNA Contamination | H6 |
| SA_00104 | RTC | Reverse Transcription Control | H7 |
| SA_00104 | RTC | Reverse Transcription Control | H8 |
| SA_00104 | RTC | Reverse Transcription Control | H9 |
| SA_00103 | PPC | Positive PCR Control | H10 |
| SA_00103 | PPC | Positive PCR Control | H11 |
| SA_00103 | PPC | Positive PCR Control | H12 |
